# Inflammatory Bowel Disease: A Stressed “Gut/Feeling”

**DOI:** 10.3390/cells8070659

**Published:** 2019-06-30

**Authors:** Yvonne Oligschlaeger, Tulasi Yadati, Tom Houben, Claudia Maria Condello Oliván, Ronit Shiri-Sverdlov

**Affiliations:** School of Nutrition & Translational Research in Metabolism (NUTRIM), Department of Molecular Genetics, Maastricht University, Universiteitssingel 50, 6229 ER Maastricht, The Netherlands

**Keywords:** IBD, gastrointestinal tract, microbiota, brain, interplay, stress

## Abstract

Inflammatory bowel disease (IBD) is a chronic and relapsing intestinal inflammatory condition, hallmarked by a disturbance in the bidirectional interaction between gut and brain. In general, the gut/brain axis involves direct and/or indirect communication via the central and enteric nervous system, host innate immune system, and particularly the gut microbiota. This complex interaction implies that IBD is a complex multifactorial disease. There is increasing evidence that stress adversely affects the gut/microbiota/brain axis by altering intestinal mucosa permeability and cytokine secretion, thereby influencing the relapse risk and disease severity of IBD. Given the recurrent nature, therapeutic strategies particularly aim at achieving and maintaining remission of the disease. Alternatively, these strategies focus on preventing permanent bowel damage and concomitant long-term complications. In this review, we discuss the gut/microbiota/brain interplay with respect to chronic inflammation of the gastrointestinal tract and particularly shed light on the role of stress. Hence, we evaluated the therapeutic impact of stress management in IBD.

## 1. Introduction

Inflammatory bowel disease (IBD) is a chronic and relapsing disorder [1], including Crohn’s disease and ulcerative colitis. While Crohn’s disease is characterized by transmural inflammation in any part of the gastrointestinal tract, ulcerative colitis is affecting the mucosal layer of the colon and rectum. Similar to other immune-mediated chronic diseases, such as rheumatoid arthritis, IBD is hallmarked by periods of remission interspersed with periods of acute flare. During disease course, symptoms like abdominal pain, cramping, loose stools or bloody diarrhea, fatigue, anemia and/or weight loss can manifest. The prevalence and incidence of IBD are increasing enormously [2]. Together with its early onset, relapsing nature, and life-threatening complications, IBD is currently a major health issue. Although the exact pathogenesis is unclear, IBD is certainly driven by disturbed crosstalk between a variety of parameters, i.e., genetic susceptibility and internal and external factors [3], which will be discussed in more detail. Although treatment options mainly focus on reducing intestinal inflammation [1], achieving/maintaining remission or improving the patient’s quality-of-life, no cure for IBD is currently available. Given that IBD is a systemic disease, often associated with comorbidities such as anxiety and depression, this narrative review aims to evaluate the mutual interplay between stress and the gut/microbiota/brain axis, particularly with regard to chronic inflammation of the gastrointestinal tract. These insights set the basis for better understanding the impact of stress management on disease activity in IBD.

## 2. Search Strategy

For this narrative review, we selected peer-reviewed preclinical and clinical articles as well as meta-analyses and important reviews from the PubMed database between January 1980 and June 2019. The following search terms were used: inflammatory bowel disease/IBD, Crohn’s disease, ulcerative colitis, irritable bowel syndrome, gut/intestines, microbiota/microbiome, brain, interplay/axis, psychological/chronic/acute/cognitive stress, lifestyle factors, stress management, stress resilience, inflammation, disease activity, therapies/interventions/therapeutic strategies.

## 3. Gut/Microbiota/Brain Interplay

In this section, we will focus on the tight association between the brain and gut, discussing the involvement of endocrine, immune, and neural pathways as well as the gut microbiota.

### 3.1. Brain/Gut Interaction

The hypothalamic pituitary adrenal (HPA) axis is an endocrine pathway belonging to the limbic system of the brain. In response to stress [4], the activated HPA axis causes the secretion of corticotropin-releasing factor (CRF) from the hypothalamus, which stimulates the pituitary gland to release adrenocorticotropic hormone (ACTH). In turn, ACTH triggers the immunosuppressive stress-hormone cortisol from the adrenal cortex [5], which ordinarily induces the synthesis of anti-inflammatory cytokines. However, in response to stress, sustained cortisol activity has also been associated with pro-inflammatory responses [6]. Likewise, stress-induced cortisol was shown to increase intestinal barrier dysfunction, as recently shown by crypt analyses from rodents and humans [7]. Moreover, administration of cortisol in a porcine model caused a shift in microbiota composition, [8], pointing towards a role for cortisol in regulating intestinal inflammation and altering microbiota composition.

In addition to the HPA axis, the autonomic nervous system (ANS) coordinates the function of the gastrointestinal tract. The ANS is known to trigger efferent signals from the central nervous system (CNS; i.e., brain and spinal cord) to the intestinal wall to regulate mucosal immune responses [9] and other intestinal functions, such as nutrient absorption [10]. Vice versa, via enteric, spinal, and vagal nerves, afferent signals from the intestinal lumen are also known to regulate behavior, sleep, and stress reactivity [11,12]. Upon receiving stimuli from the diet and gut microbiota [13,14], the enteric nervous system (ENS, “second brain”), which is part of the peripheral nervous system, mainly communicates with the CNS in a bidirectional manner. However, the ENS is also capable of intrinsically innervating the gut [15] in an autonomous manner [16].

### 3.2. Gut Microbiota

The gastrointestinal tract serves as a dynamic and local ecosystem for gut microbiota. Whereas, often being classified into two major phyla, i.e., Bacteroidetes and Firmicutes [17], the gut microbiota is composed of over 35,000 bacterial species [18]. Besides playing a role in metabolism [19], it is also essential for controlling processes related to barrier function against pathogenic microorganism colonization, such as mucosal integrity [20], immunomodulation [21], and pathogen protection [22]. Recently, preclinical [23], translational [24], and clinical [25] studies suggested that alterations in the structural composition or function of the microbiome can contribute to the development of mental illness, including depression-like behavior, and thus, is a vital component linking the gut/brain axis. In line, data have indicated strong correlations between alterations in gut microbiota and the development of multifactorial chronic inflammatory disorders, such as IBD [26,27], suggesting that dysbiosis is an important factor in both gastrointestinal and mental health.

Intestinal bacteria and their metabolites are also involved in gut-associated neuroimmune mechanisms that influence mood and behavior leading to depression. These mechanisms include tryptophan metabolism as well as neural signaling within the ENS [28]. Tryptophan is an essential amino acid, derived from the diet. While crossing the blood–brain barrier and acting as a precursor of the neurotransmitter serotonin, tryptophan can also be degraded in the gut through the kynurenine and serotonin synthesis pathways. This degradation can affect its availability to pass the blood–brain barrier. Thus, by modulating tryptophan levels, microbiota can affect the brain, resulting in behavioral changes [29].

By fermentation of dietary fibers, the gut microbiota is also responsible for producing short-chain fatty acids (SCFAs), including butyric acid, propionic acid, and acetic acid, which are typically found to be reduced in mucosa and feces of patients with IBD [30]. As extensively reviewed by Parada Venegas et al. [31], these metabolic products have shown to play an important role in promoting epithelial cell proliferation [32], barrier function [33], and cellular metabolism [34]. In addition, SCFAs have been involved in controlling intestinal inflammation through activation of G-protein coupled receptor signaling pathways [35], thereby regulating intestinal homeostasis and inhibiting pathogen colonization. Relevantly, SCFAs are also known to exert neuroprotective properties. For instance, gamma-aminobutyric acid is an inhibitory neurotransmitter involved in anxiety and depression and can therefore modulate behavior [36]. Other mechanisms by which the intestinal microbiota affect neural responses include alterations in bacterial neurometabolites or bacterial cell wall sugars. These products can either act directly on primary afferent axons or trigger epithelial cells to release molecules that modulate neural signaling within the ENS [28].

Altogether, the multifaceted interplay between the gut, microbiota, and brain allows for intestinal and extraintestinal homeostasis, thereby coordinating gastrointestinal functions and modulating mood and higher cognitive functions, respectively.

## 4. Gut/Microbiota/Brain Interplay in IBD Development

**Figure 1:**
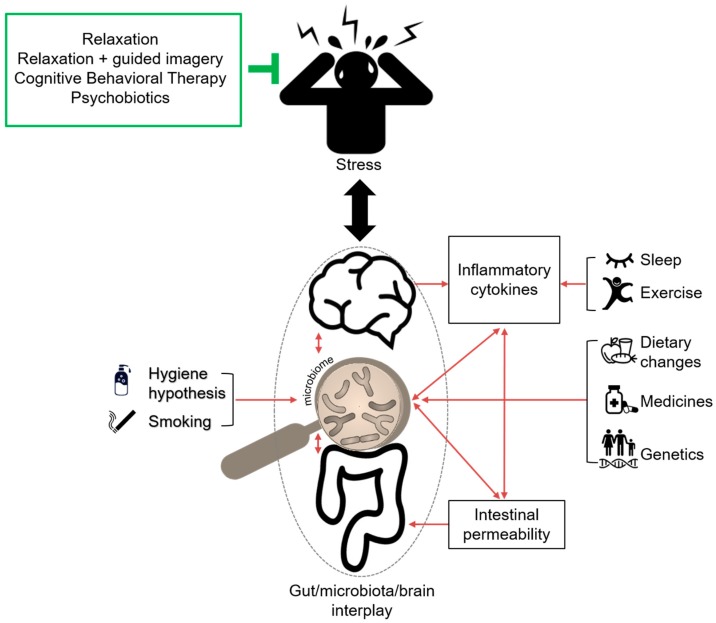
The gut/microbiota/brain interplay and its interactions upon exposure to stress. Under conditions of psychological stress including lack of sleep and physical inactivity, the brain (HPA axis) stimulates the production of pro-inflammatory cytokines. This can result in increased intestinal permeability and altered gut microbiota. In addition, fat- and sugar-enriched foods, long-term usage of medicines, as well as genetic predisposition can directly affect the gut microbiota composition, and subsequently, intestinal permeability. Furthermore, personal habits, such as hygiene and smoking, can also have an impact on the gut microbiome. Altogether, the multitude of stress-related factors can perturb the gut/microbiota/brain interplay, which contributes to the development of IBD. Relevantly, several stress management techniques have been proven to greatly alleviate IBD symptoms and improve the quality of life of IBD patients. Given that the exact underlying mechanisms in the context of IBD are not yet fully understood, therapeutic options aimed at improving stress management deserve further investigation.

It has become evident that factors such as genetics, environment, diet, and lifestyle are involved in dysregulation of the gut/microbiota/brain interplay, which in this section, will be discussed in the context of IBD development (Figure 1 and Table 1) [37,38].

To date, genome-wide association studies revealed more than 200 susceptibility gene loci in IBD [39,40,41,42,43]. First, based on a model selection analysis, 163 susceptibility gene loci were identified, of which 23 and 30 loci were shown to be specific for ulcerative colitis and Crohn’s disease, respectively [39]. These data were further completed with a more recent association study identifying 38 novel risk loci [40]. Based on another large genetic association study in IBD patients, it was shown that ten single nucleotide polymorphisms, in a total of four genes, were found to be significantly correlated with Crohn’s disease [41]. Strongest correlations were found with CYLD, a de-ubiquitinating enzyme, pointing towards the ubiquitin proteasome system as a major contributor to IBD pathogenesis [41]. A more recent and very large study of 34,819 IBD patients investigating genotype–phenotype associations across 156,154 genetic variants also provided important insights into the genetic heterogeneity between ileal and colonic Crohn’s disease, thereby rejecting the current classification of Crohn’s disease versus ulcerative colitis [42].

In addition to genetics, the association between non-coding single nucleotide polymorphisms and IBD risk has gained major interest [44]. Moreover, based on several twin studies, the importance of non-genetic environmental factors [45,46] on IBD disease manifestation has become clear. One example is the implication of micronutrients in IBD progression. Patients with IBD are commonly diagnosed with a vitamin D deficiency [47], which can be related to lowered oral intake of vitamin D or decreased sunlight exposure. A more recent study in healthy volunteers showed that, specifically, the gut microbiome of the upper gastrointestinal tract is positively influenced in response to vitamin D3 treatment [48], suggesting that vitamin D plays a protective role in IBD pathogenesis. Alternatively, intracellular levels of zinc [49] and iron [50] have been associated with bacterial clearance and consequent intestinal permeability and increased risk of IBD, respectively. 

Another environmental, lifestyle-related factor is smoking, which has been shown to cause a two-fold increased risk for IBD [51]. Besides affecting the nicotinic acetylcholine receptors present on gastrointestinal mucosal epithelial cells [52], smoking can modulate the human gut microbiota composition, thereby affecting the course of the disease in IBD. Although in the context of Crohn’s disease, smoking has adverse effects, in ulcerative colitis patients, it may play a protective role, implying that smoking may be a disease-specific modifier [53].

As mentioned, dietary fiber intake is able to prolong IBD remission through subsequent increase in luminal production of short-chain fatty acids [31,54]. In addition, it is well-established that high intake of fat- and sugar-enriched foods are capable of regulating intestinal microbiota composition and diversity [55,56], thereby also initiating and sustaining inflammation in patients with IBD [57]. Furthermore, high intake of n-3 polyunsaturated fatty acids and plant sterols have been shown to be protective [58], suggesting that dietary changes play a role in IBD pathogenesis.

It is also well known that low-to-moderate intensity exercise positively affects immune function [59]. Indeed, preclinical studies showed that moderate intensity exercise decreased the expression of pro-inflammatory cytokines, thereby improving acute colitis [60]. Human data regarding the beneficial effects of exercise on the development of intestinal inflammation are mixed, mainly due to the variations in type and rate of exercise. Several studies suggested an inversed correlation between physical activity and the risk or onset of IBD [61]; however, these effects have shown to be disease specific [62]. Nevertheless, other studies focusing on the association between exercise and disease course or the quality of life of IBD patients found a beneficial effect on well-being, sleep, confidence, and mood [63,64].

Further evidence also suggests that geographic location and socioeconomic status are associated with the risk of IBD, thereby supporting the “hygiene hypothesis” of Bloomfield [65]. This hypothesis postulates that the recent rapid rise in IBD, especially in industrialized regions [66], may be due to the lower rate of infection during childhood. The lower infection rate may evolve from reduced exposure to enteric bacteria and improved sanitation during early life [67]. Although this reasoning might indeed explain the higher incidence of IBD in urban areas, the environmental location has been shown to differently affect the prevalence of Crohn’s disease and ulcerative colitis. Whereas Crohn’s disease has shown to be more frequent in urban/coastal areas, ulcerative colitis is more prevalent in inland municipalities [68].

Given that the gut microbiota is relatively diverse and unstable during early childhood, any kind of alteration is likely to affect the intestinal immune responses and predispose individuals to IBD. For instance, medications, including antibiotics, contraceptives as well as non-steroidal anti-inflammatory drugs (NSAIDs) are known to increase the risk of IBD, likely through altering the commensal flora and/or the intestinal barrier [69]. More specifically, based on a meta-analysis, antibiotics were shown to associate with increased risk of new-onset Crohn’s disease rather than ulcerative colitis [70]. In line, a multiple database search revealed that individuals exposed to oral contraceptives had a 24% and 30% increased risk for developing Crohn’s disease and ulcerative colitis, respectively, compared with those not exposed to the medication [71]. Likewise, high doses and long-term treatments with NSAIDs [72] resulted in the exacerbation of IBD [73], potentially acting via non-selective inhibition of the cyclo-oxygenase [74].

Relevantly, IBD disease activity and its risk of relapse has also been associated with sleep disturbances [75]. Sleep disturbances can induce the levels of inflammatory cytokines, thereby activating an inflammatory cascade [76]. Furthermore, sleep disturbances have been shown to occur in IBD patients, including pediatric patients [77] as well as those with inactive disease [78], and can negatively impact quality of life. Indeed, optimized sleep duration (i.e., six to nine hours/day) was able to decrease the risk of ulcerative colitis. Further, based on a prospective study, a strong inversed correlation between sleep quality and the activity of IBD was demonstrated [79]. Also, disruptions of the circadian organization, a form of long-term biological stress, are known to affect health [80]. It has been suggested that the adverse effects of the host’s circadian rhythm, including sleep disruption, can alter the circadian rhythm of the intestinal microbiota, thereby changing its community structure [80]. Given that the gut microbiota plays a key role in the development of IBD, it is also likely that circadian disorganization, through dysbiosis of the intestinal microbiota, negatively impacts the course of the disease.

## 5. Stress and Intestinal Microbiota: Bidirectional Relationship in IBD

### 5.1. Influence of Stress on Gut Microbiota

Several lines of evidence suggest that stress, induced by dietary, environmental or neuroendocrine factors, can adversely affect the gut/microbiota/brain axis [81,82] (Table 2).

#### 5.1.1. Preclinical Studies

It was recently shown in mice that maternal nutrition can negatively affect offspring intestinal development and function. For instance, maternal high-fat diet caused a shift in microbiota composition, thereby predisposing the offspring to develop intestinal inflammation [83]. In line, early prenatal stress in rodents was shown to increase the *Oscillibacter*, *Anaerotruncus*, and *Peptococcus* genera [84] and induce a loss of *Lactobacilli* transmission to the neonate [84,85], pointing towards the involvement of birth canal delivery in gut microbiota colonization [86,87]. These data were further supported by other preclinical studies showing that short-term or mild chronic stress caused a reduction in *Lactobacilli* [88,89,90]. These data suggest that stressor-induced changes have important health implications [90]. It has also been shown, both in primates [91] and in rodents [92], that maternal separation, a form of chronic stress, was able to induce a change in fecal microbiota in new-born animals. A more recent study demonstrated that chronic stress resulted in dysbiosis of the murine gut microbiota, thereby inducing an immune system response and facilitating experimentally induced colitis [93]. Other genetically susceptible rodent models revealed that chronic psychological stress induced mucosal dysfunction, intestinal abnormalities, and subsequently intestinal inflammation [94]. Likewise, it was shown in rats that acute [95], environmental [96] as well as chronic stress [97] increased intestinal permeability, and hence, luminal molecule delivery to the mucosal immune system, thereby triggering pro-inflammatory responses. In this context, probiotics (living organisms yielding benefits on the host’s microbiome [98]) were shown to revert chronic stress-induced abnormalities of the intestinal tract [99]. Also, it was shown that diets containing a combination of specific long-chain polyunsaturated fatty acids, prebiotics, and probiotics restored the rat intestinal microbiota composition [100]. More recently, it was even demonstrated that stress hormones, through manipulation of basal corticosterone levels, were able to alter the gut microbiome of free-living birds [101]. Further, using a sophisticated rat model, water avoidance stress was shown to alter the mucus composition [102], which is known as the host’s primary innate defense. Given that changes in the production of mucosal proteins have been associated with dysbiosis of the gut microbiota, it is likely that stress indirectly affects the intestinal microbiota via inflammation of the mucosal protein layer [94].

#### 5.1.2. Clinical Studies

Few clinical studies also revealed that stress is associated with digestive problems and gastrointestinal health [103]. For instance, by means of a phylogenetic microarray, one study showed that exposure to stress during pregnancy resulted in aberrant microbiota colonization patterns in pediatrics, which likely increased inflammation and gastrointestinal symptoms [104]. In line with these findings, stress-related psychiatric disorders, such as depression, were also associated with increased bacterial translocation, thereby activating immune responses against commensal bacteria [105]. Although these data imply that stress has a potent influence on intestinal microbiota, stress is a subjective experience, which makes it challenging to objectively evaluate the effects of stress. Therefore, further human studies should be performed to verify that stress results in dysbiosis of the gut microbiota.

### 5.2. Impact of Intestinal Microbiota on Stress Responsiveness

Appropriate physiological responses to stress and/or immunity are necessary for survival. As such, aberrant responsiveness can be detrimental to the host, leading to the development of chronic disorders, including IBD [106] and brain disorders [107].

#### 5.2.1. Preclinical Studies

Preclinical studies using germ-free animals, specific pathogen-free animals, and animals exposed to pathogens, probiotics or antibiotics have been performed to better understand how gut microbiota can regulate stress response, cognition, and behavior [28]. Previously, it has become clear that intestinal colonization with conventional microbiota at an early developmental stage is important for stress responsiveness in adult mice [108]. This study showed that the HPA stress response was exaggerated in the absence of normal gut microbiota reconstitution, whereas it could be partially corrected by reconstitution of feces from specific pathogen-free mice at an early, but not at a later stage. In line with these results, other murine studies demonstrated the influence of conventional gut microbiota on the development of behavior [109,110], and showed that this effect occurred along with neurochemical changes in the brain [109]. Another study based on a rat model of acute psychological stress demonstrated that the probiotic *Lactobacillus farciminis* reduced intestinal leakiness, thereby decreasing plasma levels of lipopolysaccharides, and consequently, diminishing the HPA axis response to stress. Moreover, by reducing stress-induced plasma corticosterone levels, Bravo et al. [111] showed that the probiotic *Lactobacillus rhamnosus* was able to reduce the stress response as well as anxiety-related behaviors and cognition in mice. Furthermore, it has been shown that the combination of probiotics, such as *Lactobacillus helveticus* and *Bifidobacterium longum*, resulted in a reduction of anxiety-like behaviors in rodents [112,113].

#### 5.2.2. Clinical Studies

Messaoudi et al. [112] also validated their preclinical findings in healthy human volunteers. Their findings suggested that probiotic formulation attenuated psychological distress in healthy volunteers, which may be linked to decreased urinary free cortisol levels [112]. In agreement with these data, a randomized, double-blind, placebo-controlled trial suggested that the administration of probiotics helped in reducing anxiety-like behavior among patients with chronic fatigue syndrome [114]. Altogether, these data further confirm that the intestinal microbiota plays a role in controlling stress responsiveness, behavior, and cognition.

### 5.3. Stress and Its Impact on Inflammation

While not having discussed in detail yet, the impact of stress on the immune system appears to be quite complex. Depending on the type of stress (short-term or chronic) and/or hormones being released, a stressor may either suppress or enhance immune function [115].

#### 5.3.1. Preclinical Studies

Several preclinical studies have shown that short-term stress induces significant changes in absolute numbers and composition of blood leukocytes [116,117]. Likewise, short-term stress was shown to increase the circulating levels of interleukin-6 (IL6) and pro-inflammatory monocyte chemotactic protein-1 (MCP1/CCL2) [118]. These findings were also further confirmed by others showing that social disruption reduced microbial diversity and richness in mice, which correlated with increased circulating levels of the pro-inflammatory cytokines MCP1 and IL6 [88]. Relevantly, based on data demonstrating that administration of antibiotics was able to abolish social stress-mediated increases in pro-inflammatory cytokines [88], it is likely that intestinal microbiota plays a role in stressor-induced pro-inflammatory responses.

#### 5.3.2. Clinical Studies

Previous studies in humans provided similar evidence that stress induces an increase of pro-inflammatory Th1 cytokines [119,120,121]. For instance, academic stress, referred to as the body’s response to academic-related workload that goes beyond the adaptive capabilities of students [122], was shown to significantly increase the production of interferon-gamma (IFNγ) and tumor necrosis factor alpha (TNFα) [119]. Acute stress can also upregulate anti-inflammatory cytokines including IL10, while independently inhibiting pro-inflammatory cytokines such as TNFα [123]. These effects can induce a shift towards Th2-mediated humoral response [124], which may be essential to prevent hyperactivation of the stress system. Alternatively, chronic stress is known to increase the release of cortisol levels for several days, an effect that may be associated with immunosuppression, as shown by a reduction in circulating CD8+ lymphocytes, natural killer cells, and macrophages [125]. Nevertheless, as chronic psychological stress was also associated with increased levels of serum C-reactive protein (CRP) [126], these data suggest that chronic stress may also exert pro-inflammatory effects. Obviously, it should be noted that the gastrointestinal tract per se, including its local microbiota, may serve as an essential organ mediating immune responses [127]. This is not only of relevance in the context of human IBD, but also in irritable bowel syndrome (IBS) [128] as well as major depressive disorders [129].

### 5.4. Stress and Inflammation in IBD

Inflammatory bowel disease is a complex disease that likely does not only consist of Crohn’s disease and ulcerative colitis. Extensive translational research has been conducted to better understand the role of stress and inflammation in IBD [130].

#### 5.4.1. Preclinical Studies

Previously, using rodent models of spontaneous colitis, it was shown that intestinal inflammation is associated with defects in mucosal barrier or dysfunctional regulatory T lymphocytes [131]. Other studies using mice fed a dextran–sulfate–sodium diet to induce colitis revealed the importance of intestinal adhesion molecules, such as ICAM-1, in the development of intestinal inflammation [132]. Relevantly, when dextran–sulfate–sodium-treated mice were injected with enterotoxigenic *Bacteroides fragilis*, increased colitis and colonic inflammation was observed [133]. Similarly, in cellular models, *Clostridium difficile* toxin A was shown to induce apoptosis and inflammation in enterocytes [134,135]. Moreover, using a mouse model of depression, it was shown that stress-induced release of corticosteroids can reactivate IBD [136], likely via increased production of pro-inflammatory cytokines. These data imply that stress may affect the course of the disease.

#### 5.4.2. Clinical Studies

In humans, it was shown that infections with *Bacteroides fragilis*, through secretion of its pro-inflammatory toxin, is associated with development of ulcerative colitis [137]. Likewise, infections with *Clostridium difficile* is thought to be involved in the reactivation of IBD in patients [138]. Within a similar context, it was also shown that patients with ileal Crohn’s disease had a higher percentage of invasive *Escherichia coli* in the mucosa as compared to healthy controls, and these percentages even correlated with severity of the disease [139]. In line, another study demonstrated that *Escherichia coli* can replicate inside macrophages of patients with Crohn’s disease and subsequently secrete large amounts of TNFα, thereby contributing to inflammation [140]. Collectively, these data point towards the strong relationship between the gut microbiota composition and intestinal inflammation.

It is also important to note that psychosocial stress, including psychological distress, anxiety, and depression, can induce low-grade chronic inflammation in the gut. For instance, in humans, it was shown that depression correlated with elevated levels of TNFα and CRP, pro-inflammatory cytokines that that are known to trigger inflammation in patients with IBD [141,142]. Hence, it has been proposed that stress gradually contributes to the development or exacerbation of IBD (as reviewed elsewhere [28,143]). Vice versa, compared to the general population, young patients with IBD displayed higher rates of psychological stress, and these results were further confirmed in gastrointestinal disorders such as IBS [144,145]. Together, these data imply that the disease itself may have a direct impact on the quality of life of patients.

Yet, it is not clear whether individuals with higher stress also experience more IBD symptoms. A large cross-sectional, population-based study of IBD patients showed that the relationship between intestinal inflammation and symptomatic disease activity differed between Crohn’s disease and ulcerative colitis [146]. Whereas Crohn’s disease did not associate with intestinal inflammation and disease activity symptoms, an association was found for ulcerative colitis. These findings suggest that the duration and intensity of stress factors may have differential influences on chronic inflammatory diseases. Furthermore, this study showed that perceived stress in both diseases correlated with disease activity symptoms, while not with inflammation [146]. Although the majority of conventional therapies in IBD focus on tackling intestinal inflammation, these insights open new venues for stress reduction in the management of IBD.

## 6. Managing Stress in IBD: Does It Make the Gut Feel Better?

In recent years, significant progress has been made in the treatment of IBD, focusing either on targeted therapies [147] or on alternative and complementary strategies [148], which has recently been extensively reviewed [147,148]. Nevertheless, there is no certain cure for IBD due to the limited effectiveness of current therapies, which often even goes hand in hand with significant side effects. Quality of life as well as anxiety and depression are known predictors of negative medical outcomes in many chronic conditions, and as reviewed, stress has a profound impact on these variables in patients with IBD. Hence, a number of approaches have focused on relieving stress as a potential therapeutic option in IBD (Table 3) [148,149].

Stress management is a technique used to diminish the physiological effects of stress and tension, and to help the individual to improve his/her coping skill. One variant of such therapy is relaxation, by which the individual is trained by a therapist or in a self-directed manner to create physiological and mental rest. Several studies showed the effectiveness of relaxation training in a variety of physical illnesses, including cardiovascular disease, arthritis [150], and IBD [151,152,153]. Previously, it was shown that stress management could significantly improve IBD symptoms such as pain and fatigue [151]. In line, behavioral self-management therapy resulted in a 57% decrease in 1 year risk to relapse in IBD patients [152], supporting the beneficial effects of stress management in IBD. More recently, two clinical studies pointed towards the beneficial effects of mindfulness on psychological and physical symptoms, quality of life, and C-reactive protein, an established biomarker [154], in patients with IBD [155,156]. However, based on a systematic review, McCombie et al. [153] concluded that the effect of psychotherapy led to inconsistent results in IBD patients. It should be noted that the studies included in this study focused on a wide range of therapies. Thus, although meditation and relaxation may have beneficial effects on inflammatory activity and quality of life in IBD patients, the effectiveness of mindfulness-based interventions on disease activity remains to be elucidated.

Whereas several studies demonstrated that psychotherapy had a beneficial impact on anxiety [157], depression [158], and quality of life of IBD patients [159], other studies were not able to find any effect [160,161]. Yet, two other studies investigated the impact of combining a variety of techniques as a treatment option in IBD [161,162]. Indeed, when combining relaxation with guided imagery, a method focusing on mind-relaxing images to replace stressful thoughts, both anxiety status and quality of life appeared to be improved among patients with IBD [163]. Similarly, multi-convergent therapy, which combines mindfulness meditation with cognitive behavioral therapy, has been used as a therapeutic option in patients with tinnitus and IBS. Therefore, its applicability and efficacy were investigated in an IBD population that received conventional therapy [164]. This study revealed that multi-convergent therapy improved quality of life mainly in IBD patients suffering from IBS-like symptoms [164], suggesting that this strategy has beneficial effects only in a subgroup of IBD patients. Although IBS and IBD are medically distinct from each other, symptoms compatible with IBS indeed often co-exist in patients with IBD [165], and should therefore not be underestimated. Within this context, it was recently shown that 36% and 37% of CD and UC patients, respectively, met IBS diagnostic criteria [165], confirming that the presence of IBS-like symptoms in IBD is common [148]. Furthermore, patients with IBS in quiescent IBD were shown to have significantly more anxiety and depression than patients without IBS [165].

More recent data suggested that short-term cognitive behavioral therapy improved quality of life and depression scores in patients with IBD, while not affecting disease activity or other measures of psychological well-being [166]. Others particularly investigated the impact of mind–body therapy, a combination of moderate exercise, diet, stress management training, behavioral techniques and self-care strategies on patients with ulcerative colitis. Results suggested that this approach has a positive effect on IBD development by improving quality of life and mental/physical health scores [167].

As reviewed in detail elsewhere, other complementary and alternative medicines, such as herbal medicine, vitamin supplementation, and exercise have also gained attention for its anti-inflammatory properties and usefulness in the treatment of IBD [148,168]. Within this context, preclinical data from mice suggested that cannabinoid receptor activation mediates protective mechanisms in experimental colitis [169,170]. In line with these data, two clinical studies reported that cannabis was able to reduce IBD symptoms [171,172], pointing towards its ability to treat IBD. Nevertheless, whether cannabis is able to positively affect the course of disease requires further investigation. Other preclinical studies in mice showed an important role for vitamin D and its receptor in the regulation of inflammation of the gastrointestinal tract [173]. Indeed, IBD patients often lack this vitamin [47], and hence, studies investigated the role of vitamin D [174,175,176] in treatment of IBD patients. Although these studies pointed towards beneficial effects on disease activity and risk of relapse, other clinical studies on the use of vitamin B [177] or K [178] in IBD treatment were inconsistent. These data imply that there is a lack of evidence to support positive effects of vitamins on IBD disease course [148]. Likewise, whereas preclinical [60] and clinical studies [179,180] suggested that low-to-moderate intensity exercise exerted beneficial effects on intestinal inflammation, overall health, and quality of life of IBD patients, further studies investigating the impact of exercise on disease activity and/or prevention of IBD are warranted [148].

Given that stress orchestrates an important influence on structural and functional aspects of the microbiome, multiple studies have also investigated the role of psychobiotics in stress-related diseases. Psychobiotics refer to probiotics or prebiotics that can manipulate commensal gut microbiota, and when ingested at adequate quantities, may indirectly have positive psychiatric effects in psychopathology [181]. As extensively reviewed, both in experimental colitis and human IBD, pre- and probiotics have shown beneficial effects in the prevention of IBD by modulating the trophic functions of the microbiota, improving the intestinal mucosal barrier and mediating anti-inflammatory responses [182]. Given that the intake of psychobiotics also seem to exert antidepressant effects, including improvements in mood and decreases in stress-related plasma and urinary free cortisol [181], it may be postulated that psychobiotics might serve as therapeutic modulators of the gut/microbiota axis and positively influence psychological functions in the context of IBD.

Altogether, the quality of life and course of IBD are regulated by psychological conditions, and as such, the implementation of stress management may play an important role in IBD disease regression (see also Figure 1). Nevertheless, current studies are limited in sample size and study design, and hence, may lack proper controls. Therefore, future research is needed to validate current treatment options and/or to explore novel therapeutic opportunities in order to prevent IBD onset or improve stress-affected well-being of IBD patients.

## 7. Conclusions

In summary, this review summarized the tight connection between the gut, microbiota, and brain in the context of IBD and has particularly shed light on the impact of stress on this interplay. It should be noted, however, that it is rather challenging to investigate the impact of stress on IBD, as stress can arise from totally different origins and may be closely connected to potential individual confounding factors, including (mental) health status and inter-individual variability in stress responsiveness and/or vulnerability. Therefore, future research involving preclinical studies as well as large-scale, controlled clinical trials should not only focus on unravelling the exact mechanisms through which stress affects IBD. However, it is also of great interest to further investigate the exact mechanisms of how stress management can orchestrate beneficial effects in IBD and how stress-relieving therapies should be implemented in IBD care.

## Figures and Tables

**Table 1:** Factors involved in the gut/microbiota/brain interplay in inflammatory bowel disease (IBD) development.

Factor	Type of study	N	Intervention/Methodology	Outcome	Author(s)	Reference
**Genetics**	Clinical study	637	Questionnaire	First-degree relatives have 10-fold increased risk of IBD development	Orholm, M. et al.	37
	Meta-analysis	>75,000 cases & controls	GWAS	Identified 30 gene loci for CD and 23 for UC	Jostins, L. et al.	39
	Trans-ancestry association studies	238,401	GWAS	Identified 38 susceptibility loci for IBD	Liu, J.Z. et al.	40
	Genetic association study	6228	Association studies	Identified UPS and CYLD gene are important in IBD pathogenesis	Cleynen, I. et al.	41
	Genotype association study	34,819	Association studies	Insights into genetic heterogeneity between ileal and colonic CD	Cleynen, I. et al.	42
	Clinical study	323	Array-based transcriptome data	Identified 99 strong positional candidate genes in 63 risk loci	Momozawa, Y. et al.	43
	Clinical study	189 twin pairs	Questionnaire	Results highlight the importance of environmental triggers	Spehlmann, ME. et al.	45
	Clinical study	80 twin pairs	Questionnaire	Genetic influence is stronger in CD than in UC	Halfvarson, J. et al.	46
**Diet**	Interventional, open-label, pilot study	16	Vitamin D3 supplementation	Vitamin D3 modulates the gut microbiome	Bashir, M. et al.	48
	In-vivo mouse study	4–8 mice per group	Oral antibiotics	Increased levels of intracellular zinc led to bacterial clearance	Lahiri, A. et al.	49
	In-vivo mouse study	4–5 mice per group	High-fat diet and oral antibiotics	High-fat diet alters gut microbiome composition	Hildebrandt, MA.	55
	In-vivo mouse study	>100 inbred mouse strains	High-fat/high-sucrose diet	High-fat/high-sucrose diet influences gut microbiota composition	Parks, BW. et al.	56
	Case-control study	86	Dietary intake	Mono- and polyunsaturated fats consumption is a risk factor for IBD	Geerling, BJ. et al.	57
	In-vivo mouse study	4–5 mice per group	Diet enriched with phytosterols	Phytosterols are protective against IBD	Aldini, R. et al.	58
**Environment**	Population-based case-control study	1382	Questionnaire on 25 different topics	Altered intestinal microbiota may modulate risk of IBD	Ng, SC. et al.	62
	Retrospective case study	1194	Clinical history and questionnaire	Higher prevalence of CD in urban areas and UC in inland areas	Carpio, D. et al.	68
**Smoking**	Meta-analysis	245 articles	Smoking	Smoking is a risk factor for IBD	Mahid, SS. et al.	51
	Prospective case-control study	160	Transdermal nicotine or placebo patches	Smoking effects gut microbiota composition	Richardson, CE. et al.	52
**Medication**	Meta-analysis	11 observational studies	Antibiotic exposure	Antibiotics increases the risk of new-onset CD than UC	Ungaro, R. et al.	70
	Meta-analysis	20 studies	OCP	Increased risk for development of CD and UC	Ortizo, R. et al.	71
	Case-control study	122	NSAIDs	Provoked disease activity in IBD	Felder, JB. et al.	72
**Exercise**	In-vivo mouse study	4 mice per group	Exercise training	Alleviated symptoms of acute colitis	Saxena, A. et al.	60
	Prospective cohort study	194,711	Physical activity	Inversely associated with risk of CD	Khalili, H.	61
	Uncontrolled pilot study	12	12-week walking program	Beneficial for IBD patients	Loudon, CP. et al.	63
**Sleep disturbances**	Longitudinal,internet-based cohort data	3173	Questionnaire	Increased risk of disease flares in CD but not UC	Ananthakrishnan, AN. et al.	75
	Clinical study	10	Disturbances in sleep-wake cycle	Sleep disturbances led to immunologic alterations	Born, J. et al.	76
	Clinical study	47	Questionnaire assessing sleep quality	Impaired sleep quality is associated with pediatric IBD	Mahlmann, L. et al.	77
	Clinical study	32	Questionnaire assessing sleep quality	Impaired quality of life in IBD	Keefer, L. et al.	78
	Prospective observational cohort study	41	Pittsburgh sleep quality index (PSQI)	Strong association between poor sleep quality and IBD	Ali, T. et al.	79
	In-vivo mouse study	33	Diet and sleep disturbances	Circadian disorganization impacts intestinal microbiota	Voigt, RM. et al.	80

CD = Crohn’s disease; GWAS = genome-wide association studies; NSAIDs = nonsteroidal anti-inflammatory drugs; OCP = oral contraceptive pill; SNP = single nucleotide polymorphism; UC = ulcerative colitis; UPS = ubiquitin protease system.

**Table 2:** Studies investigating the link between stress and gut microbiota.

Factor	Type of Study	N	Intervention/Methodology	Outcome	Author(s)	Reference
**Prenatal/early life stress**	In-vivo mouse study	6–20 mice per group	Maternal high-fat diet	Dysbiosis and low-grade inflammation in the intestine	Xie, R. et al.	83
	In-vivo rat study	6–10 per group	Prenatal stress	Long-lasting alterations in the intestinal microbiota composition	Golubeva, AV. et al.	84
	In-vivo mouse study	21–23 mice per group	Prenatal stress	Alterations in vaginal microbiota contributed to reprogramming of the developing brain	Jasarevic, E. et al.	85
	In vivo primates study	20	Maternal separation	Maternal separation-induced psychological disturbances altered intestinal microflora	Bailey, MT. et al.	91
	In-vivo rat study	22	Maternal separation	Early life stress induced alterations in gut-brain axis contributing to IBD symptoms	O’Mahony, SM. et al.	92
	Longitudinal clinical study	192 children	Questionnaire	Prenatal stress is associated with microbial colonization patterns in infants	Zijlmans, MA. et al.	104
**Chronic/social/environmental stress**	In-vivo mouse study	7–20 mice per group	Chronic social defeat	Stress induced complex structural changes in the gut microbiota	Bharwani, A. et al.	81
	In-vivo mouse study	10 mice per group	Lactation	Cellular transfer of bacterial translocation occurred in pregnant and lactating mice	Donnet-Hughes, A. et al.	87
	In-vivo mouse study	5 mice per group	SDR	Stress led to significant changes in intestinal microbiota colonization	Bailey, MT. et al.	88
	In-vivo mouse study	10–12 (3 independent experiments)	Unpredictable chronic mild stress	Altered intestinal microbiota composition, specifically the *lactobacillus* compartment	Marin, IA. et al.	89
	In-vivo mouse study	5 mice per group	SDR	Affected microbial populations that are closely associated with the colonic mucosa	Galley, JD. et al.	90
	In-vivo mouse study	4–6 mice per group	Chronic restraint stress	Disturbed gut microbiota and subsequent activation of immune system led to colitis	Gao, X. et al.	93
	In-vivo rat study	7–8 rats per group	WAS	Intestinal inflammation by impaired mucosal defenses against luminal bacteria	Soderholm, JD. et al.	94
	In-vivo rat study	6	Cold-restraint stress or WAS	Exacerbated intestinal inflammation due to increased uptake of immunogenic substances	Saunders, PR. et al.	95
	In-vivo rat study	*not specified*	Stress induction	Increased gastrointestinal permeability, allowing luminal constituents to the mucosal immune system	Meddings, JB. et al.	96
	In-vivo rat study	4 rats per group	WAS	Stress-induced epithelial mitochondrial damage and mucosal mast cell activation	Santos, J. et al.	97
	Field experiment in wild birds	64	Corticosterone-implant	Altered gut microbiome in free-living birds	Noguera, JC. et al.	101
	In-vivo rat study	13–14 rats per group	WAS	Altered intestinal mucus composition	Da Silva, S et al.	102
	In-vivo mouse study	18–24 mice per group	Germ-free and specific-pathogen free; acute restraint stress	Commensal microbiota can affect the postnatal development of the HPA stress response	Sudo, N. et al.	108
	In-vivo mouse study	12 mice per group	Germ-free and specific-pathogen free	Conventional intestinal microbiota influenced the development of behavior	Neufeld, KM. et al.	109
	In-vivo mouse study	7–14 mice per group	Germ-free and specific-pathogen free	Gut microbiota affected mammalian brain development and subsequent adult behavior	Diaz Heijtz, R. et al.	110
	Clinical study	263	Daily interview assessment	Stress is associated with digestive problems and gastrointestinal health	Walker, LS. et al.	103
	Clinical study	40	Depression	Increased bacterial translocation and activated immune responses against commensal bacteria	Maes, M. et al.	105
	Clinical study	65	Coping style instrument	IBD adolescents used more avoidant coping styles compared to healthy controls	Van der Zaag-Loonen, HJ. et al.	106
**Pro/prebiotics**	In-vivo rat study	4–5 rats per group	WAS and probiotics	Probiotics prevented chronic stress-induced intestinal abnormalities	Zareie, M. et al.	99
	In-vivo rat study	84	Maternal separationand prebiotics/probiotics/LC-PUFA	Nutritional intervention at weaning normalized gut permeability and restored growth rate	Garcia-Rodenas, CL. et al.	100
	In-vivo mouse study	36	Probiotic formulation	Suggested the importance of probiotics in gut/brain axis in stress-related disorders	Bravo, JA. et al.	111
	In-vivo mouse study	8 mice per group	Chronic mild stress and probiotics	Decreased pro-inflammatory cytokines and altered stress-related behaviors	Li, N. et al.	113
	In-vivo rat study	36 rats;	Probiotic formulation	Anxiolytic-like activity in rats	Messaoudi, M. et al.	112
	Double-blind, placebo-controlled, randomized parallel group study	66 individuals	Probiotic formulation	Beneficial psychological effects in healthy human volunteers	Messaoudi, M. et al.	112
	Systematic review	11 RCTs	Prebiotic supplementation	Short-term beneficial effects in intestinal microbiota composition	Rao, S. et al.	114

HPA = hypothalamic pituitary adrenal axis; LC-PUFA = long-chain poly-unsaturated fatty acids; RCT = randomized controlled trials; SDR = social disruption; WAS = water-avoidance stress.

**Table 3:** Clinical studies investigating stress management in IBD.

Factor	Type of Study	N	Intervention	Outcome in IBD Patients	Author(s)	Reference
**Guided imagery/Relaxation training**	Pilot RCT	28	Guided imagery with relaxation (GIR)	Improved QL in elderly women with osteoarthritis	Baird, CL. et al.	150
	Prospective RCT	39	Relaxation-training	Beneficial effects on anxiety, pain and stress in IBD patients	Mizrahi, MC. et al.	163
**Self-directed stress management**	Clinical study	45	3 types of stress management, including self-directed and conventional medical treatment	Trained CD patients showed reduced fatigue, constipation and abdominal pain, whereas no beneficial effects in conventional-treated CD patients	García-Vega, E. et al.	151
**Lifestyle management**	Clinical study	60	60-h training program in lifestyle modification over a period of 10 weeks	Short-term benefits in the QL in UC patients, whereas no long-term effects	Langhorst, J. et al.	157
	Clinical study	49	8-session information about QL and stress management	No effect on anxiety levels 6 months post-intervention	Larsson, K. et al.	160
	Prospective, randomized waiting-control group design	30	60-h training program on life style management	Improved QL in patients with UC remission	Elsenbruch, S. et al.	167
	Prospective, randomized study	32	Low-intensity walking program	Improved QL of CD patients	Ng, V. et al.	179
	Prospective RCT	30	Moderate-intensity running	Improved QL of IBD patients	Klare, P. et al.	180
**Psychotherapy**	Two clinical trials	36	7-session behavioral protocol	57% reduction in IBD relapse in the following 12 months	Keefer, L. et al.	152
	RCT	41	Primary and Secondary Control Enhancement Therapy-Physical Illness	Beneficial effects on depression in IBD adolescents	Szigethy, E. et al.	158
	Clinical study	178	Nurse-led counselling	Improved QL over 6 rather than 12 months in IBD patients	Smith, GD. et al.	161
	Prospective, uncontrolled open trial	30	Supportive-expressive group psychotherapy	No changes in QL, anxiety, or depression over the course of treatment in UC/CD	Maunder, RG. et al.	162
	Meta-analysis	1824 studies with 14 RCTs	Psychological therapy	Small short-term beneficial effects on QL and depression in IBD patients	Gracie, DJ. et al.	166
	RCT	29	Breath-Body-Mind Workshop; questionnaire	Significant long-lasting benefits for IBD symptoms, anxiety, depression and QL	Gerbarg, PL. et al.	155
	Control study	60	Mindfulness-based stress reduction	Improved mood and QL after six months of intervention	Neilson, K. et al.	156
	RCT	36	Gut-directed hypnotherapy	Gut-directed hypnotherapy may be one aspect in a disease-management program for IBD	Keefer, L. et al.	159
	Clinical trial	66	Multi-convergent therapy (psychotherapy)	Therapy is beneficial in the management of IBD symptoms	Berrill, JW. et al.	164
**Medication**	Retrospective observational study	30	Herbal treatment	Positive effect of cannabis on disease activity in CD	Naftali, T. et al.	171
	Prospective, placebo-controlled study	21	Herbal treatment	Short course of cannabis had beneficial effects in CD patients	Naftali, T. et al.	172
	Double-blind RCT	108	Placebo or vitamin D3	Reduced relapse risk in CD	Jorgensen, SP. et al.	174
	Prospective	37	Active or plain vitamin D	Active form of vitamin D has short-term beneficial effects in CD	Miheller, P. et al.	175
	Meta-analysis	12 studies	Serum folate and vitamin B12	Low concentration of serum folate is a risk factor for IBD and supplementation may be beneficial	Pan, Y. et al.	177
	Double-blind RCT	10 per group	Placebo/ phylloquinone/ vitamin D3	No significant beneficial effects of phylloquinone on bone health in CD patients	O’Connor EM. et al.	178

CD = Crohn’s disease; QL = quality of life; RCT = randomised controlled trial; UC = ulcerative colitis.

## Abbreviations

ACTH	Adrenocorticotropic hormone
ANS	Autonomic nervous system
CNS	Central nervous system
CRF	Corticotropin-releasing factor
CRP	C-reactive protein
ENS	Enteric nervous system
HPA	Hypothalamic pituitary adrenal
IBD	Inflammatory bowel disease
IBS	Irritable bowel syndrome
IFNγ	Interferon-gamma
IL	Interleukin
NSAIDs	Non-steroidal anti-inflammatory drugs
MCP1	Monocyte chemotactic protein-1
SCFA	Short-chain fatty acids
TNFα	Tumor necrosis factor alpha
